# Genetic Diversity of Armenian Grapevine (*Vitis vinifera* L.) Germplasm: Molecular Characterization and Parentage Analysis

**DOI:** 10.3390/biology10121279

**Published:** 2021-12-06

**Authors:** Kristine Margaryan, Gagik Melyan, Franco Röckel, Reinhard Töpfer, Erika Maul

**Affiliations:** 1 Research Group of Plant Genetics and Immunology, Institute of Molecular Biology of National Academy of Sciences RA, Yerevan 0014, Armenia; 2 Department of Genetics and Cytology, Yerevan State University, Yerevan 0025, Armenia; 3 Voskehat Educational and Research Center of Enology, Branch of Armenian National Agrarian University, Merdzavan 1139, Armavir Province, Armenia; 4 Julius Kühn-Institute (JKI), Institute for Grapevine Breeding Geilweilerhof, 76833 Siebeldingen, Germany; franco.roeckel@julius-kuehn.de (F.R.); reinhard.toepfer@julius-kuehn.de (R.T.); erika.maul@julius-kuehn.de (E.M.)

**Keywords:** grapevine, *Vitis vinifera*, Armenia, genetic diversity, parentage analysis, nSSR markers

## Abstract

**Simple Summary:**

The knowledge of genetic diversity and relatedness among grapevine varieties is important for recognizing gene pools. One of the major goals of the present large-scale study was to evaluate the level and relationships of existing genetic diversity across Armenia, aiming to identify genotypes that could provide genetic insights into the Armenian grapevine germplasm structure. A combination of nuclear microsatellite markers and ampelography proved useful to determine the identity of collected samples recovered from old vineyards and home gardens. Synonyms, homonyms, alternative spellings, and misnomers were clarified. First-degree genetic relationships between autochthonous varieties were partly uncovered. Missing parents might still exist in old vineyards but were not sampled yet or might have disappeared over time. The continuation of prospections to fill that gap is planned. The high number of new bred varieties included in the study reflects the enormous breeding activity in Armenia. The high number of alleles, high level of observed and effective heterozygosity, and presence of female APT3-allele 366, which is absent in western European cultivars, illustrate the huge diversity of the Armenian germplasm. Presumably, these findings are related to recurrent introgression of *Vitis sylvestris* into the cultivated compartment during domestication events. So far, the present study is the first most representative and comprehensive analysis of Armenian grape germplasm.

**Abstract:**

Armenia is an important country of origin of cultivated *Vitis vinifera* subsp. *vinifera* and wild *Vitis vinifera* subsp. *sylvestris* and has played a key role in the long history of grape cultivation in the Southern Caucasus. The existence of immense grapevine biodiversity in a small territory is strongly linked with unique relief and diverse climate conditions assembled with millennium-lasting cultural and historical context. In the present in-depth study using 25 nSSR markers, 492 samples collected in old vineyards, home gardens, and private collections were genotyped. For verification of cultivar identity, the symbiotic approach combining genotypic and phenotypic characterization for each genotype was carried out. The study provided 221 unique varieties, including 5 mutants, from which 66 were widely grown, neglected or minor autochthonous grapevine varieties, 49 turned out to be new bred cultivars created within the national breeding programs mainly during Soviet Era and 34 were non-Armenian varieties with different countries of origin. No references and corresponding genetic profiles existed for 67 genotypes. Parentage analysis was performed inferring 62 trios with 53 out of them having not been previously reported and 185 half-kinships. Instability of grapevine cultivars was detected, showing allelic variants, with three and in rare cases four alleles at one loci. Obtained results have great importance and revealed that Armenia conserved an extensive grape genetic diversity despite geographical isolation and low material exchange. This gene pool richness represents a huge reservoir of under-explored genetic diversity.

## 1. Introduction

In the *Vitis* genus, which consists of nearly 60 inter-fertile species, *Vitis vinifera* is the only species indigenous to Eurasia and is suggested to have first appeared ~65 million years ago [1]. Within *Vitis vinifera*, two subspecies exist: *V. vinifera* subsp. *sylvestris* including the wild populations and *V. vinifera* subsp. *vinifera*, resulting from domestication of the wild progenitor and including about ten thousand cultivated varieties [2,3]. Grapevine belongs to the most important domesticated fruit crops. Few other species are historically and economically so important as *Vitis vinifera* spp. *vinifera*. According to the International Organization of Vine and Wine (OIV) the global grape area is estimated at 7.4 million hectares, with a production of 77.8 million tons of fresh grapes and 260 million hectoliters of wine (http://www.oiv.int, accessed on 15 August 2021).

Archaeological, historical, and genetic studies reveal that South Caucasus, Northern Iran and Southeast Anatolia are most likely the places of earliest grape domestication [4,5]. There is evidence of human habitation for more than twenty thousand years in the mountains of southern Caucasus and transitional types of grapes, including *V. vinifera* subsp. *sylvestris* and feral types, cultivated, and ancient indigenous varieties are common in this region [6]. The process of grapevine domestication generated modifications in the biology and architecture of the grape plant [1]. The main modifications were the appearance of hermaphroditic flowers in *V. vinifera* subsp. *vinifera*, an increased number of berries per cluster, a higher sugar content, the enlargement of bunch and berry size, seedlessness in table grapes (generally through stenospermocarpy and only rarely via parthenocarpy) and the change in seed morphology, which is the most stable character to differentiate remains of wild or cultivated grape [7,8,9].

Domestication of grapevines occurred nearly eight thousand years ago, during the Neolithic Age. After that, the migration of cultivars took place along with the spreading of wine culture from their primary domestication center in the Near East to Mesopotamia, Levant, Africa, and Europe [10,11,12,13]. Recently, researchers applied SOM (self-organizing maps) portrayal, a neural network-based machine learning method, with the aim of re-analyzing the genome-wide single nucleotide polymorphism (SNP) data of 783 grapevine cultivars collected from Middle Asia to the Iberian Peninsula and from overseas regions [14,15]. Based on the obtained results, genomic landscape and the different sample similarity plots were consistent with the historical knowledge and mirror the geographical distribution of grape varieties, indicated main pathways of grape dissemination and genome-phenotype associations about grape usage. According to SOM analysis, cultivated grapes occurred initially in South Caucasus and Fertile Crescent (South East Anatolia, North Lebanon and Syria) and then disseminated towards the Mediterranean world to the West and into the East towards Iran and the Middle East (Tajikistan, Uzbekistan), Afghanistan, and India. The northern and southern ways into the west agree with the distribution of settlements of Greeks and Phoenicians, respectively [15].

The World Wide Fund for Nature defined the Caucasus region as a Global 200 Ecoregion, based on criteria such as species richness, high levels of endemism, taxonomic uniqueness, and global rarity of major habitat types [16]. The Republic of Armenia is located in the South-Central Caucasus on the crossroad of the biogeographic zones of the Lower Caucasus, the Iranian, and Mediterranean areas and includes many regionally endemic, relict and rare species. The country is of particular importance as a center of endemism for wild relatives of economically important crops, including grapevine. Armenia is a unique grapevine diversity hotspot in the South Caucasus where viticulture and winemaking are dating back to the beginning of the IV Millennium BC [17]. The three main drivers (sexual reproduction, vegetative propagation, and spontaneous mutations) had a considerable impact on the enrichment of genetic diversity in Armenia and the long-standing history of grape cultivation accounts for the existence of a wide range of indigenous varieties. Together with abundantly thriving wild species, they are well adapted to the different eco-geographic and climatic conditions of the country [18].

Viticulture and wine industry in particular always played a vital role as the most important sectors of the Armenian economy, and the production of brandy is one of the main branches of its export. Wine production became industrialized already in the 1870s when Nerses Tairyan/Tairov started industrial production of vodka. Later, from 1887 on brandy production progressed on the territory of the former Yerevan fortress. During the pre- and Soviet Era, grape-growing and winemaking were leading branches of agriculture in Armenia and strongly connected to the development of capitalism and market demands in Russia. In the 1975’s the vineyard surface planned to be enlarged and reach peak levels up to 80–90 thousand ha. At that time, Armenia accounted for 72 percent of all brandy production in the South Caucasus despite its small territory [19,20]. The industrial development of wine, vodka and brandy production promoted the development and profitability of the viticultural sector in the country. More than 850 grape varieties, including 400 autochthonous varieties, were preserved in the first Armenian National Grapevine Collection established in the 1950s at the Institute of Wine-Making and Fruit-Growing [21]. The living collection was entirely destroyed after the USSR collapsed in the early 1990s and ten years later three new ampelographic collections were established, preserving 140 accessions, from which only 70 were native Armenian varieties. Due to the economic and political situation, the maintenance of the Armenian grapevine germplasm in these three collections was also stopped.

**Figure 1:**
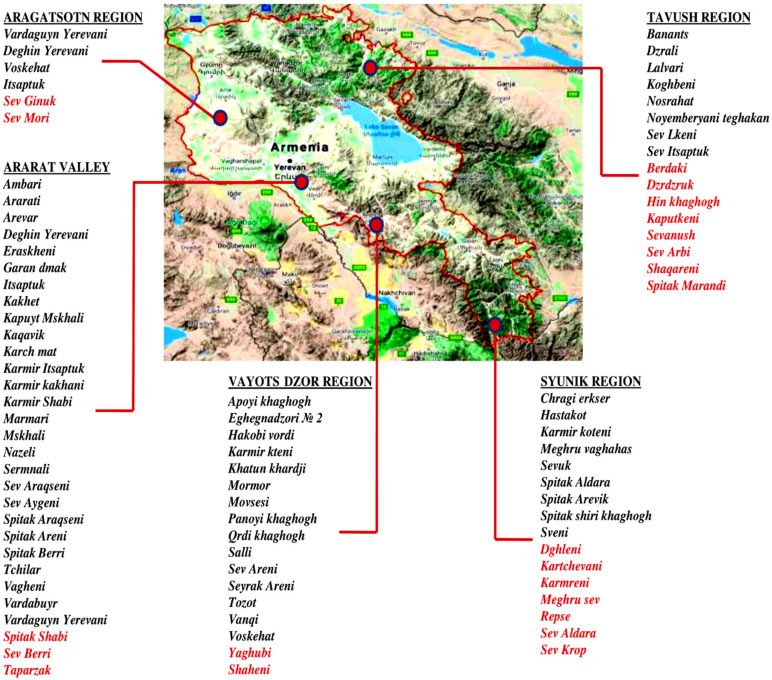
The most iconic indigenous grapevine varieties of viticulture regions of the Republic of Armenia. The red marked varieties were not rediscovered yet.

In 2016, with the support of Food and Agriculture Organization (FAO), a nationwide program to collect, conserve and characterize Armenian grapevine germplasm was conducted. In the newly established Armenian National Grapevine Collection in Etchmiadzin 293 grapevine accessions were planted. On the map of the Republic of Armenia, viticultural regions and their most iconic grapevine varieties native for each area are presented in Figure 1.

The precise number of Armenian varieties is not clearly known and there is often uncertainty about their real number since synonyms and homonyms occur. The rescue and conservation of grapevine genetic diversity in Armenia are particularly urgent since a large number of local native varieties are no longer cultivated or existing as single vines and are therefore at serious risk of loss.

During the last years in parallel with wine industry development, only a limited number of varieties were used for wine production with a serious shift to single variety vineyards. The intensive cultivation of a small number of commercial cultivars has resulted in an alarming reduction in genetic diversity since only about 30 from 400 native grape varieties are used in wine and brandy production. Minor autochthonous cultivars having only a local significance in the different wine-growing regions are under-exploited. Their ignorance might be related to the lack of comprehensive characterization of native neglected varieties, especially to missing data on oenological and agronomical traits and partially, due to demands of the wine/brandy market. All of these arguments prove the necessity and importance of collection, conservation, characterization, and efficient use of grape germplasm resources, as well as knowledge of genetic diversity and genetic relationships between genotypes [22].

Until recently, the only method traditionally used in Armenia for the characterization and identification of grapevine varieties was ampelography (Aµπϵλος, “vine” and γραφος, “writing”) a method based on morphological description mainly of shoot-tips, leaves, bunches, and berries [23]. Ampelography is an accurate and reliable method, but factors such as subjectivity, variability related to vineyard management, climate, and sanitary status sometimes preclude unambiguous identification. However, trueness to type assessment of grape varieties is required in viticulture, research and for effective germplasm management. In addition, clarification of synonyms, homonyms and misnomers is essential. Nuclear simple sequence repeats (nSSRs) or microsatellites are the most appropriate and efficient markers which are co-dominant, highly polymorphic, and easily transferable across related *Vitis* species. They are widely used primarily for the differentiation and identification of cultivars, parentage analysis, and genetic mapping [24,25,26,27,28,29,30].

Very little is known about the magnitude of grape germplasm in Caucasian countries, although this region is considered a center of grape diversity [4]. To fill that gap, a large-scale investigation of Armenian grapevine genetic resources via molecular characterization in complement with morphology was carried out. The objectives of the projected work were: (i) recovery and safeguard of Armenian germplasm; (ii) determination of the grapevines identity; (iii) detection of synonymies, homonymies and misnaming; (iv) assessment of the level of genetic diversity; and (v) investigation of genetic relatedness of Armenian grape germplasm. For verification of cultivar identity, the symbiotic approach combining genotypic and phenotypic characterization for each genotype was carried out. Genetic characterization was performed by 25 nSSR markers. Phenotypic studies involved the comparison of morphological features with existing bibliographies and online databases, such as the *Vitis* International Variety Catalogue (*V*IVC) (https://www.vivc.de/, 15 August 2021) and Réseau Français des Conservatoires de Vigne (https://bioweb.supagro.inra.fr/collections_vigne/SearchS.php, 15 August 2021).

The presented research was part of the extensive research started in 2017 in the scope of the Armenian-German bilateral project intending preservation, promotion, and prominence of native grape germplasm towards recovering the untapped genetic diversity of *V. vinifera* in Armenia [31].

## 2. Material and Methods

### 2.1. Plant Material

Prospections were carried out in five traditional viticultural regions of Armenia: Ararat Valley (Ararat and Armavir districts), Aragatsotn, Vayots Dzor, Tavush, and Syuniq during the vegetation and harvest period. Sampling regions are reported in Figure 1. The nationwide survey mainly focused on vineyards established in the 1900s and earlier; some of them were totally out of cultivation. Family gardens were included, as well as a few small private collections. More than one hundred twenty locations were retained. Vines were selected through the support of local industry members and vineyard owners. Variety designations (if known) and vine age were recorded. Genotypes lacking a designation were named taking into account morphological traits, village or wine grower’s names or viticultural areas. GPS coordinates and elevations of the sampled accessions were registered. A total of 492 samples were collected for molecular fingerprinting.

### 2.2. DNA Extraction and nSSR Analysis

Total genomic DNA was extracted from 100 mg of young leaf tissue after grinding with MM 300 Mixer Mill system (Retsch, Haan, Germany) and stored at −80 °C until use. DNA extraction was performed employing the DNeasy 96 plant mini kit (QIAGEN, Dusseldorf, Germany) following to the manufacturer’s protocol. DNA concentration and quality were checked by spectrophotometric analysis and electrophoresis in 1% agarose gel. Microsatellite fingerprinting of genotypes were performed on 24 microsatellite loci (nSSRs) well distributed across the nineteen grape chromosomes as previously described [32,33] (i.e., VVS2, VVMD5, VVMD7, VVMD21, VVMD24, VVMD25, VVMD28, VVMD27, VVMD32, four of the VrZAG series (VrZAG62, VrZAG79, VrZAG67, VrZAG83), VMC4f3.1, VMC1b11 and nine of the VVI series VVIb01, VVIn16, VVIh54, VVIn73, VVIp31, VVIp60, VVIv37, VVIv67, and VVIq52). Nine polymorphic microsatellite markers proposed by the GrapeGen06 (http://www.montpellier.inra.fr/grapegen06, 15 August 2021) project: VVMD5, VVMD7, VVMD25, VVMD27, VVMD28, VVMD32, VVS2, VrZAG62, and VrZAG79 were used for comparison of genetic profiles with the SSR-marker database of the Julius Kühn-Institut (JKI), maintaining about eight genetic profiles from distinct sources. Fingerprints from the European *Vitis* Database (www.eu-vitis.de, 15 August 2021) produced during European project GrapeGen06 and resulting from COST Action FA1003 were used to find corresponding profiles [34,35,36]. Fifteen additional markers were applied for parent-offspring analysis.

For fragment length determination by capillary electrophoresis on ABI 3130xl Genetic Analyzer (Applied Biosystems, Life Technologies, Waltham, MA, USA), all forward primers were 5′-labelled with a fluorescent dye (FAM, HEX, TAMRA, ROX and PET). The combination of markers with different labels and diverse fragment lengths allows one to perform the polymerase chain reaction (PCR) and grouped markers in seven multiplex pools, comprising two to five SSR markers characterized by similar annealing temperatures (Supplementary Table S1). The 2x KAPA2G Fast PCR Kit (Duren, Germany) was used to set up 5 μL reaction mixtures containing master mix, 100 pmol of each primer and 1 ng of template DNA. GeneAmp PCR system 9700 thermal cycler (Applied Biosystems, Schwerte, Germany) was used for the amplification starting with 3 min initial denaturation at 95 °C, followed by 30 cycles for 30 s. A final extension was performed at 72 °C for 7 min. 1 μL of the PCR product was used for fragment length determination and the results were processed with GeneMapper 4.0 software (Applied Biosystems, Life Technologies, Waltham, MA, USA) recorded in base pairs. Allele size was determined by comparing the fragment peaks with the internal size standard, using the Microsatellite default method for size calling with SSR and the expected repeat size. To correct the amplification shifts among different multiplexes, SSR profiles were adapted by including in each PCR amplification run the DNA of standard cultivars Cabernet franc and Muscat à Petits grains blancs.

### 2.3. Flower Phenotype Analysis

The determination of flower sex was carried out for all genotypes collected throughout Armenia and was analyzed by a specifically designed APT3 marker from adenine phosphoribosyl transferase gene capable to distinguish flower sex: female (F), male (M) or hermaphrodites (H) [37].

### 2.4. Genetic Diversity Analysis

The genetic diversity among grapevine genotypes was estimated. The standardized nSSR genotyping data were used to determine the number of different alleles (Na), the number of effective alleles (Ne), Shannon’s Information Index (I), observed heterozygosity (Ho), expected heterozygosity (He, and fixation index (F) referred to as the inbreeding coefficient. The allele frequency for each nSSR loci was calculated as well. The GenAlEx software version 6.5 was used to compute genetic diversity statistics for each nSSR locus [38,39]. Clustering was performed by MEGA 7 software, version 7.0.26, which was used to generate a distance tree by the Neighbor-joining (N-J) hierarchical clustering method [40,41] based on the pairwise euclidean distance created from the genetic distance obtained in GeneAlEx 6.5 software.

### 2.5. Parentage Analysis

Parentage analysis was carried out by Cervus 3.0.7 using non-redundant grapevine genotypes to determine possible first-order kinship relationships: trios (mother-father-offspring) and duos (parent-offspring pairs) using the likelihood-based method implemented by software [42]. Based on bibliographic information and breeder’s records 44 additional candidate parents of Armenian and non-Armenian origin were involved in parentage analysis to validate the provided information. The likelihood of each detected trio and duo was evaluated considering the natural logarithm of the overall likelihood ratio (LOD) score with a higher confidence level (>95%), obtained by simulation, was used as the criterion for parentage assignment. A maximum number of mismatching loci was one nSSR for duos and trios, respectively.

Colony software (version 2.0.6.6) was applied to reconstruct genotypes used by Armenian breeders, but not available in the data set. Colony parameters were set up in order to perform one medium-length run with the full likelihood (FL) method, allelic dropout rate 0, and other genotyping error rates 0.0050 [43]. Inferred genotypes were included in parentage analysis to discover further possible relationships.

## 3. Results

The solid determination of trueness to type of grapevine genotypes required a combination of molecular fingerprinting, morphological description, and exhaustive bibliographic studies. The identity of each genotype was defined based on the analysis of 25 SSR markers and comparison of genetic profiles with almost eight thousand fingerprints documented in the JKI-SSR-marker database, the European *Vitis* Database and genetic profiles generated during COST Action FA1003 and bibliography. Three volumes of Armenian ampelographies (published in 1947, 1962, and 1981), three volumes of Russian ampelographies (published in 1946–1956, 1963–1970, and 1984), Caucasus and Northern Black Sea Region Ampelography (published in 2012) and Ampelography by Melyan et al. (published in 2019), represented the most important sources [19,44,45,46,47,48,49,50]. Analysis of collected Armenian gemplasm revealed the following main cases: (i) synonyms (different cultivar names, but identical fingerprints), (ii) homonyms (identical or very similar cultivar names, but different fingerprints), (iii) unique genotypes, and (iv) questionable accessions (confirmed trueness to type based on ampelographic descriptors. However, varietal status based on SSR profile is questionable, and there are obvious differences between morphological descriptions in bibliography and the accessions features in the vineyard).

An overview of the huge information gathered is displayed in Supplementary Table S2, including *V*IVC variety number, prime name, Armenian variety name, accession numbers of varieties preserved in Armenian national grapevine collection, origin/pedigree given by breeder, color of berry skin, flower sex phenotype, flower sex genotype (ATP3 marker), utilization, viticulturual region, the status of identity, confirmation of variety based on leaf and bunch morphology, number of collected samples, bibliographic references for genetic profile identification and ampelographic references used for confirmation of trueness to type. The numbers of bibliographic references correspond to source codes in *V*IVC (www.vivc.de, accessed on 15 August 2021). Owing to the fact that no living references were available, morphological features of the analyzed varieties were compared with descriptions and photographs available in bibliographical references.

### 3.1. Identification of Prospected Material

A total of 492 genotypes were analyzed using 25 SSR-markers, revealing 216 unique profiles, with 271 genotypes being duplicate individuals such as synonyms, homonyms, or clones. Five mutants were identified, from which four are autochthonous Armenian grape varieties: Mormor, a berry color chimaera of Sev Areni, Marmari/Kishmish mrarmornyi, a chlorophyllic mutant, Vardaguyn Yerevani/Kishmish rozovyii a berry color mutant of Sultanina and Kishmish chernyi teinturier, a berry flesh color mutant of Sev Qishmish/Kishmish chernyi variety. In addition, a black berry color mutant of Muscat à petits grains blancs, sampled in an old vineyard was determined. Among the 216 unique profiles 66 were old autochthonous, 49 were new bred Armenian cultivars, and 34 were non-Armenian varieties with different countries of origin (Supplementary Table S2). No references and corresponding genetic profiles existed for 67 genotypes (Supplementary Table S2). Twenty-five genotypes were identified via ampelographic references and a new variety name was assigned to twenty genotypes, based on morphological characteristics and cultivated area. Three arguments lead to the assignment of new names: more than one vine of the same genotype existed in the set, distinct sampling sites and genetic relationships.

Among analyzed genotypes the most frequently found grape varieties were Sev Areni (27 samples), Tozot (18 samples), Karmir kakhani (15 samples), Rizamat (10 samples), Apoyi khaghogh (9 samples) and Spitak Arevik (9 samples). Sev Areni and Tozot are the most emblematic ancient wine grape varieties and displayed a wide range of intravarietal clonal diversity. Apoyi khaghogh is one of the neglected grape varieties and is preserved only in few private vineyards in Vayots Dzor region. Karmir kakhani and Spitak Arevik are old autochthonous table grape varieties. All samples collected as Karmir Shabi, described in ampelography as an old table grape variety, matched the genetic profile of Rizamat.

From the set of 271 redundant samples, a few cases illustrating variety identification, synonyms and homonyms, and both new named and unknown genotypes are presented in Supplementary Table S3. Redundant samples, except mutants, were excluded from further analysis. Genetic profiles are provided in Supplementary Table S4.

### 3.2. Flower Phenotype

Flower phenotype was analyzed by APT3 marker capable to distinguish females from hermaphrodites or male plants. According to the obtained data among 221 distinct varieties, eight different allelic patterns were determined at APT3 loci: 27 genotypes have shown 268/268 (F), 14 genotypes 268/397 (F), 6 genotypes 268/336 (F), 1 genotype 336/336 (F), 54 genotypes 268/397/466 (H), 97 genotypes 268/466 (H), 21 genotypes 336/466 (H), and 1 genotype 268/336/466 (H) alleles. Thus, among analyzed genotypes 173 were hermaphrodite and 48 turned out to be female.

Field phenotypic observations were carried out during the flowering period and matched flower phenotypes predicted by DNA analysis (Supplementary Table S2).

### 3.3. Assessment of Genetic Diversity and Relationships of Armenian Grape Germplasm by Microsatellite Analysis

Genetic data from 24 nuclear microsatellites across 221 unique grapevine varieties of *V. vinifera* subsp. vinifera were used in the present study. The range of allele size (Ra), number of different alleles (Na), effective number of alleles (Ne), Shannon’s information index (I), observation heterozygosity (Ho), expected heterozygosity (He), and fixation index (F) were calculated to assess the genetic diversity of Armenian grape germplasm. Statistics about the discriminatory efficiency of the 24 SSR markers are presented in Table 1. The high number of different alleles (347) proves the high degree of observed genetic variability.

The number of alleles per SSR locus ranged from 5 (VVIn16) to 25 (VVMD 28) and the mean allele number per loci was 14.485. The effective number of alleles, respecting alleles that occur at a relevant frequency within the sample, ranged from 2.035 for locus VVIn73 to 10.241 for locus VMC4f3.1, with a mean of 5.531. The following loci displayed high Ne values as well: VVIv37, VVIp31, VVS2 and VrZAG67. The highest Shannon’s information index (I) was observed in VMC4f3.1 locus (2.548) and lowest in VVIn73 (0.999), while the average among SSR loci was 1.9. Shannon’s information index is an important parameter mirroring the level of polymorphism. For microsatellite markers efficiency observed and expected heterozygosity (Ho, He) are considered to evaluate the genetic variability among analyzed varieties. The observed heterozygosity (Ho) ranged from 0.548 (VVIn73) to 0.914 (VVIp31), with an overall mean of 0.787. The expected heterozygosity (He) values ranged between 0.509 (VVIn73) to 0.902 (VMC4f3.1), with an average of 0.789. The overall mean of observed and expected heterozygosity was almost the same. Fixation index (F), a parameter reflecting a reduction in heterozygosity level and thus an indicator of inbreeding ranged between −0.099 (VVIp31) to 0.149 (VVIh54) with a mean value of 0. The observed negative F values indicated an abundance of heterozygote genotypes presuming random mating.

Allele size (AS) and frequencies (AF) for each of the microsatellites are presented in Supplementary Table S5. The majority of analyzed loci have shown at least one allele with a frequency higher than 0.20. For VVMD32, VVIv67, VrZAG83, VVIn16, VVIp60, VVMD24, VVMD21, VVIb01, VVIh54, and VVIq52 alleles with a frequency range from 0.30 to 0.40 was observed. For the nine polymorphic microsatellite markers proposed by GrapeGen06 the most frequent alleles were determined: VVS2 (133, 143 bp), VVMD5 (236, 238 bp), VVMD7 (239, 249 bp), VVMD25 (241, 249 bp) VVMD27 (186, 195 bp), VVMD32 (250, 272 bp), VrZAG62 (188, 196 bp), and for VrZAG79 (247, 251 bp).

**Figure 2:**
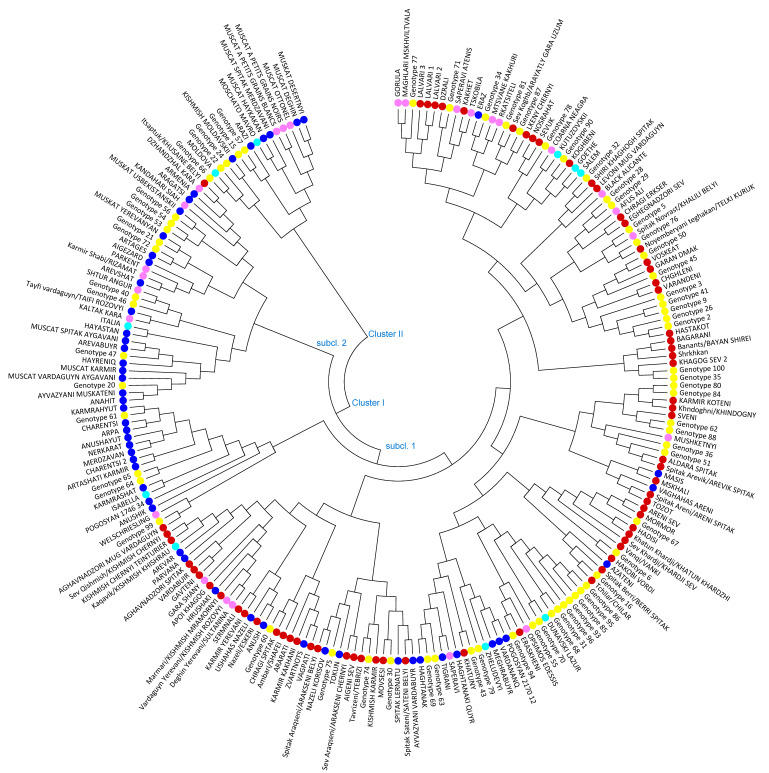
Neighbor-joining dendrogram showing genetic relationships among 221 Armenian grapevine varieties based on 24 SSR loci. Armenian autochthonous varieties are marked with red, Armenian new bred cultivars with blue, unknown individuals with yellow, non-Armenian autochthonous varieties with rose and non-Armenian new bred cultivars with light blue colour.

The neighbor-joining (NJ) distance tree was constructed to study genetic relationships among the 221 grape varieties based on allele frequencies of 24 SSR loci. Two major clusters with subclusters were distinguished grouping native-known varieties together with new bred varieties and unknown, respectively non-identified vines and evidenced genetic relationships among analyzed grapevine genotypes (Figure 2). Cluster I with 2 main sub-clusters encompasses 205 grapevine accessions, including the most ancient Armenian wine grape varieties, new bred cultivars and 62 unknown genotypes out of 67 involved in the analysis. One hundred seventy-two grapevine varieties were grouped in the first subcluster of Cluster I, comprising the most iconic wine grape varieties Sev Areni, Tozot, Sev khardji, Hadisi, Vanqi, Hakobi vordi, Eraskheni, Voskehat and the oldest table grape varieties such as Garan dmak, Spitak Aldara, Spitak Arevik, Spitak Sateni, Spitak Araqseni, Sev Araqseni, and the majority of Qishmish varieties, which are clearly separated. The second subcluster of Cluster I combined 33 grapevine varieties, among which 9 are unknown individuals, 7 varieties are non-Armenian with a different country of origin, 16 are Armenian new bred cultivars and Itsaptuk/Khusaine belyi is the only autochthonous Armenian variety, progenitor of new bred Aragatsi and Armenia cultivars. Cluster II is a blend of only 16 genotypes, encompasses six Armenian and five non-Armenian varieties and five unknown genotypes. Within the cluster seven Muscat varieties are clearly distinguished.

### 3.4. Parentage Analysis

Parentage analysis was carried out with 24 nSSR markers of 265 non-redundant grapevine genotypes. To increase the discovery of first-order kinship relationships, profiles of forty-four varieties of Armenian and non-Armenian origin were included from Lacombe et al., 2013 and the JKI-microsatellite database. Their choice was based on country of origin is Armenia and previously stated relatedness to Armenian germplasm [51]. Colony was used to infer missing progenitors of crosses. Based on records of Pogosyan, the breeder of almost 100 Armenian varieties, genetic profiles of Seyanets 3-14-15, Seyanets 1563, Seyanets S 484, Seyanets S 1262 and the Inferred genotype 14 were reconstructed and included in parentage analysis.

In Supplementary Table S6 proposed full parentages (mother, father and offspring) of Armenian grape varieties are presented, including LOD values, the number of loci and comments. According to the obtained results, 62 trios were detected (zero mismatching loci for all genotypes) with 53 out of them having not been previously reported. From proposed trios, breeders’ data were confirmed for 25 new bred cultivars. Parentage analysis allowed also to discover half confirmation cases for 7 varieties, where for 2 cases new P1 (mother) and for 5 cases new P2 (father) were identified. For Csarenci and Spitak Lernatu P1 and P2 were identified and for Anushik and Hayreniq breeders’ data were invalidated and new parent candidates were suggested. In this respect it is important to note, that phenotypic characterization of these varieties perfectly matched with ampelographic descriptions. Among 62 full parentages for 15 varieties information about original crosses did not exist and trios were constructed for the first time. In addition, we identified P1 and P2 for nine unknown genotypes and three new named varieties. Concerning the nine autochthonous grape varieties, trios of seven varieties were proposed for the first time and two trios confirmed prior publication. Our results confidently support that Hadisi, one of the emblematic wine grape varieties in Armenia, was the progenitor of the presumably oldest varieties Spitak Berri, Chilar and Khatun khardji. Obtained results revealed that among new bred cultivars the most common parents were Karmrahyut, Angur Kalan, Seyanets S 1262 and Muscat Hamburg, widely used by Armenian breeders especially during Soviet Era.

In addition, 185 half kinships (varieties sharing at least one allele at each of the 24 nSSR loci) were found and duos are presented in Supplementary Table S7. According to the obtained results following cases of relationships were found: (i) 28 cases of putative first-degree relationships (PO, with no possibility to determine if a variety is a parent, an offspring, or a full sibling of the second variety), out of which 21 relationships are reported for the first time; (ii) for 79 out of 185 duos the breeders’ data were confirmed and for 15 duos breeders data were invalidated; (iii) for 22 duos first-degree relationships were confirmed by prior publications and (iv) 24 duos the status as putative new crosses were confirmed.

Based on parentage analysis the varieties, which were frequently involved in Armenian germplasm by first-order relationship, were defined: Angur Kalan, Madeleine Angevine, Muscat Hamburg, Katta Kurgan, Karmrahyut, Seyanets S1262, Seyanets S484, and Hadisi.

## 4. Discussion

### 4.1. Recent History

Armenia is an important center of origin both for cultivated *Vitis vinifera* subsp. *vinifera* and wild *Vitis vinifera* subsp. *sylvestris* and played an essential role in the long history of grape cultivation in the Southern Caucasus [16]. The existence of huge grapevine biodiversity in a small territory such as Armenia is strongly linked with unique relief and diverse climate conditions assembled with millennium-lasting cultural and historical context. In the present extensive in-depth study, the analyzed set of grapevine genetic diversity included widely grown and neglected autochthonous and minor grapevine varieties, as well as new bred cultivars created within the national breeding programs mainly during Soviet Era.

During the last twenty years, nuclear microsatellite markers, as efficient molecular tools and powerful complement of ampelographic characteristics, were prevalently applied for management of the *Vitis* genetic resources, such as identification of accessions and parentage analysis, solving problems and uncertainties related to homonymy, synonymy, misnaming, and inferring the genetic structure of grape populations [52]. The prevalence of synonymous and homonymous cultivars within the Armenian grape germplasm has been described and reported before in the scope of the first large scale survey realized by the cooperation of Armenian-German researchers [31]. The described cases of synonyms and homonyms usually are related to migration events often linked with an alteration of names. The management of grape genetic resources is a complex issue that requires technical, agronomic and scientific efforts, as accurate authentication, documentation and registration of genetic resources. Uncertainties within grape germplasm in terms of synonymous and homonymous varieties existed practically in each grape-growing country. In Armenia, it became more complicated since in 1947 by the decision of the National Academy of Sciences of Armenia, the name of 76 native grape varieties were replaced with new analogues, with the aim to reflect their Armenian origin (Table 2) [44]. The old names of these varieties remained in ampelographies as synonyms. However, until now in some of the viticultural regions, local farmers are using mainly the old names instead of the new ones. This fact often is the main reason for uncertainties affecting also accurate documentation and registration of the correct number of Armenian grape varieties in bibliographies and databases.

The first Armenian Ampelography, which includes precise morphological, agro-biological, and technological characteristics of local and foreign grapevine varieties was published in 1947 [44]. In the first volume, 16 local standard and 42 local non-standard varieties were described, as well as 19 foreign/imported varieties as Aleatico, Aligote, Riesling, Saperavi etc. The second volume published in 1962 by S.H. Poghosyan includes a description for 71 neglected local varieties, 13 unknown varieties, 31 new bred varieties and 24 prospective elite seedlings [19]. The third volume was published already in 1981 again by S.H. Poghosyan, encompassing characterization of 34 less known/unknown local grapevine varieties, 39 new bred varieties, 11 table and 14 wine elite seedlings [45]. Recently two further ampelographies were added—Caucasus and Northern Black Sea Region Ampelography, published in 2012, providing descriptions of 34 local Armenian varieties and Ampelography, published in 2019 by G.G. Melyan, including 55 autochthonous varieties and 34 new bred cultivars [49,50].

The present comprehensive research on grapevine genetic resources, prospected throughout traditional viticultural regions across Armenia, allowed to examine and sometimes revise available descriptions found in ampelographies, providing a more rigorous and newer prospect on the origins and relatedness of grapevine varieties in Armenia. Obtained results have great importance and revealed that Armenia preserved a wide range of grape genetic diversity despite geographical isolation of the country and low material exchange, which was also suggested by COST Action FA1003 [36]. This gene pool richness represents a huge reservoir of under-explored genetic diversity.

### 4.2. Genetic Diversity and Relatedness in the Armenian Germplasm

The knowledge of genetic diversity and relatedness among grapevine varieties is important to recognize gene pools. One of the major goals of the present large-scale study was to evaluate the level and relatedness of existing genetic diversity across Armenia, aiming to identify genotypes that could provide genetic insights into the Armenian grapevine germplasm structure. It turned out that Armenian germplasm is a blend of different genotypes, exhibiting a high level of differentiation, resulting in higher-than-expected levels of heterozygosity. This is often observed in woody perennial crops where varieties are selected for their vigor and crop performance, indirectly endorsing high levels of heterozygosity [53,54]. Eastern European varieties displayed the largest non-biased heterozygosity and largest number of common and private alleles in a dataset of 2096 single profiles from the Vassal-Montpellier collection proving this finding [55].

In the present study, the majority of the clones within a variety revealed no difference and were grouped as one variety cluster when all 492 individuals were subjected to neighbor-joining cluster analysis (cluster not provided). However, some of them such as Sev Areni, Nazeli, Garan Dmak, and Mskhali underwent somatic mutations and showed varying genotypes and, to some extent, varying phenotypes analogous [56,57]. Observed variations of SSR length can occur naturally during vine growth mainly due to diverse types of mutations, which are responsible for intravarietal diversity. Clones displaying enough divergent traits are considered as different varieties. Diversity level of clones within varieties seem to depend on the age and the spreading of the variety. Ancient varieties exposed to environmental stresses during a long period of time can accumulate a comparably high level of mutations [58,59,60]. A special case was autochthonous Lalvari with three types of observed allelic variations at VVMD7 (249–259, 233–247 and 247–259) and VVIV37 (160–162, 160–170, 156–164). Several varieties, such as Khatun Khardji, Charentsi, Spitak Lernatu, and Hastakot appeared to be three-allelic at one microsatellite locus, revealing chimerism [61,62]. This phenomenon was illustrated in a study of Velez [63].

During the process of Armenian grapevine germplasm analysis, we have found cases, when genetically related varieties have shown very similar morphology and variety identification based only on visual observation was hard and cases, when clones of varieties noticeably differed in morphology without a change in the genetic fingerprints.

The application of microsatellites as highly polymorphic molecular markers is a proven efficient tool describing the level of heterozygosity across and within grapevine varieties. In the present study, the neighbor-joining cluster analysis based on 24 SSRs was applied without considering the geographic origin of the genotypes. However, the analyzed set clearly demonstrated an obviously structured arrangement of samples according to geographic origin, particular characteristics such as muscat flavor or seedlessness and ancestry. Genetic relationships between autochthonous and new bred varieties were elucidated, as well as cases related to unknown respectively not described genotypes.

A majority of the identified varieties were grouped in Cluster I including Armenian native and new bred cultivars, non-Armenian varieties and unknown genotypes. In Cluster I the ancient indigenous grape varieties Lalvari, Dzrali, Sev Lkeni, Sev Koghb/Arayatly gara uzum, originated from Tavush, a region next to the Georgian border, as well as Kakhet, Eraz and unknown genotypes 71, 34, 81, 87 were combined with Gorula, Maghlari Mskhviltvala, Saperavi Atenis, Mtsvane kakhuri and Rkatsiteli, native to Georgia. According to parentage analysis Sev Lkeni displayed PO relationship with Sev Koghb/Arayatly gara uzum and unknown genotype 87. For unknown genotype 81 Sev Koghb/Arayatly gara uzum and Rkatsiteli were proposed as parents. New bred cultivars in Cluster I are Eraz, an interspecific cross having in pedigree Rkatsiteli, Kutuzovski (Coarna neagra x Dattier de St. Vallier), whose pedigree given by the breeder was confirmed and the introduced American varieties Goethe and Salem. Carter x Schiava grossa as indicated progenitors of Goethe and Salem were confirmed. Carter is most likely extinct. However, Colony inferred the missing genotype based on nine presumed Carter offspring (data not published).

Cluster analysis revealed genetic similarity among the old autochthonous grapevine cultivars Shiri khaghogh spitak, Levoni mug vardaguyn (named variety), Chragi Erkser, Eghegnadzori sev (named variety), Spitak Novrast/Kalili Belyi, Noyemberyani teghakan/Telki Kuruk, Voskehat, Garan Dmak, Varandeni, Chghleni, Hastakot and unknown genotypes 32, 28, 29, 5, 76, 50, 45, 3, 41, 9, 26, and 2 (consecutive order in the cluster). Surprisingly, Afus Ali (Lebanon) and Black Alicante (Spain) were involved in the group. Those non-Armenian varieties were sampled from private old vineyards. PO relationships were found among Hastakot and unknown genotype 2 and genotype 26. Hastakot is an old autochthonous Armenian variety and was never used in crosses by Armenian breeders. Thus, genotypes 2 and 26 are considered being ancient Armenian varieties, which identity could not be determined yet. The further bibliographical investigation is needed. The most iconic Voskehat and Garan Dmak, autochthonous grapes, have shown PO relatedness with Hadisi. PO relationship of Voskeat and Hadisi confirmed the finding of Lacombe et al. [51]. Chragi Erkser is an endangered autochthonous red wine grape variety. During survey, four different samples Sev Chragi, Chragi Erkser, Chragi lavik, and Spitak Chragi were collected. According to molecular fingerprinting Sev Chragi corresponded to Sev Areni and Chragi lavik matched Chragi Erkser (Supplementary Table S3). Spitak Chragi with white berries was a no name vine. It was designated Spitak Chragi, since it shared similar morphologic traits with Chragi Erkser, except berry color. Effectively parentage analysis revealed that Spitak Chragi is a progeny of Chragi Erkser and unknown genotype 60.

One of the cases that required clarification is related to Kalili Belyi (Spitak Khalili) variety. According to the first book of Armenian Ampelography by Tumanyan one of the recorded synonyms of Spitak Sateni is Spitak Khalili (spitak means white) [44]. Spitak Sateni is an old endangered table grape, cultivated mainly in Ararat Valley. During field prospections, the sample named Spitak Sateni was collected from a very old vineyard in Elpin village, Vayots Dzor region. It matched to the genetic profile of Khalili Belyi. The genetic profile of Vaghahas Eghia, collected from Ararat Valley corresponded to Aushon Rannii from the Bulgarian *Vitis* Database [64]. In 2019 a genetic fingerprint of Spitak Sateni was obtained from Vassal-Montpellier collection (https://www6.montpellier.inrae.fr/vassal_eng/, 15 August 2021) [32]. It turned out to be matching Aushon Rannii, respectively, Vaghahas Eghia. Further detailed ampelographic investigation and comparison of leaf and bunch photos with bibliographic references and herbarium leaves of Vassal-Montpellier collection confirmed that finding and that Aushon Rannii of the Bulgarian *Vitis* Database can be considered as a case of a misnaming. According to the Russian Ampelography Khalili Belyi is known under different designations in post-Soviet Union countries: in Turkmenistan Ak Khalili, in Dagestan Ay izyum, in Astrakhan region of RF Tsarskiy and in Armenia Novrast beliy/Spitak Novrast. According to the authors, Khalili belyi originated from Iran. The homonym Khalili is fairly abundant, varieties differ in morphology and biological characteristics, but are similar with respect to their early ripening time [46]. Thus, based on molecular fingerprinting and morphological description, Spitak Sateni and Spitak Novrast/Kalili Belyi are distinct varieties and cannot be considered as synonyms. It is important to note, Tumanyan describing Sev and Spitak Sateni and Sev and Spitak Araqseni underlined the relatedness of these varieties based on close morphology and emphasized the probability of berry color mutation. According to our comprehensive analysis, combining ampelography and molecular fingerprinting by 24 nSSR markers, we confirmed the distinct identity of Spitak Sateni, Sev Araqseni, and Spitak Araqseni and that these genotypes cannot be considered as berry color mutants. However, Spitak Sateni and Sev Araqseni turned out to be PO related and Sev Sateni was not recovered yet.

Clustering allowed grouping of indigenous wine grapes Karmir Koteni, Khndoghni, Sveni, Spitak Areni, Tozot with autochthonous table grapes Spitak Aldara, Spitak Arevik and Mskhali. In this group also unknown genotypes 80, 84, 62, 88, 36, 51 were arranged together with two new bred cultivars Masis and Vaghahas Areni and Mushketnyi from Russia. This group perfectly mirrored the geographic distribution of indigenous wine and table grapes Karmir Koteni, Khndogni and Sveni and Spitak Aldara and Spitak Arevik, respectively, with all the unknown genotypes. All these samples were collected from Syuniq region and Artsakh. For Karmir Koteni and Khndoghni first-order relationships were not found. Parentage analysis revealed PO relationships between unknown genotypes 62 and 88. In Ampelography by Poghosyan [19] and Aivazyan [65] authors mentioned Sveni as one of the synonyms of Khndoghni. This is a true case of homonymy since ampelographic description, genetic fingerprinting, and literature analysis demonstrated that Sveni and Khndoghni are distinct varieties. Spitak Areni turned out to be the progeny of Tozot and Sev Areni. The use of the same designation “Areni” indicates that similarity of morphological traits exists, which viticulturists recognized in former times. It is important to note, that according to bibliography, Spitak Areni is hermaphrodite. However, for all collected seven samples, female phenotype was stated. APT3 marker analysis for all samples has shown 268–336 genotypes. Phenotypic variability related to bunch architecture of this variety from very loose to very dense was observed.

Unexpectedly, the collected samples Karmir khach, Gyogyam, Itsitstseni, Karch mat, Karmir milagh, Salli, and Tozot showed matching genetic profiles. Gyogyam, Itsitstseni, Karch mat, Salli, and Tozot were described as distinct varieties in ampelographies. They all shared close morphologic characteristics such as circular, five-lobed leaves, conical, dense or very dense bunches and black, slightly elliptic/ovate berries covered by dense/middle dense layer of fax. The comprehensive comparison of bibliography and ampelographic features led to the final conclusion that these varieties are clones of Tozot. Karmir khach is a locally known name in Aghavnadzor village, Vayots Dzor region. The accession collected as Karmir milagh was a case of misnaming, since Karmir milagh is a documented synonym of Karmir kakhani.

In Cluster I the most ancient and emblematic Armenian grape varieties Sev Areni, Mormor, Hadisi, Khatun Khardji, Sev Khardji, Vanqi, and Hakobi vordi collected from Vayots Dzor region and Spitak Berri and Chilar collected from Ararat Valley were grouped together with unknown genotypes 67, 6, and 16. The oldest and well-preserved industrial wine complex was discovered in 2007 in Areni village, Vayots Dzor region dating back to the end of the 5th and the first quarter of the 4th millennium B.C [66]. The ancient winemaking facility with a platform for crushing grapes and karases (pithoi for storing wine) evidence 6000 years of a winemaking tradition in this area.

Parentage analysis, unexpectedly, underlined the key role of Hadisi participating in revealed trios and duos. Poghosyan [19] did a detailed description of this variety. A single plant was found by local farmers in Hadis Mountain at 1650 m above sea level located in the Kotayk region. Based on morphology the farmer suggested the variety originated from cultivated grape and most likely seeds were brought by birds. In two perfect trios, Hadisi and unknown Genotype 16 revealed being genitors of Spitak Berri and Chilar. Poghosyan and Melyan described Spitak Berri as an old autochthonous grape cultivated in Etchmiadzin, Ashatarak and Artashat [19,50]. One of the synonyms of Spiatk Berri is Spitak Orduchi Chilar, most likely based on morphological similarities shared with Chilar. Both of them are characterized by oblong cylindrical-conical, rarely branched bunches and elliptic or ovate, green-yellowish berries. Tumanyan and Melyan described Chilar in detail [44,50]. The variety is considered very ancient and is cultivated in Artashat and Kotayq regions. One of the synonyms of Chilar referred by Tumanyan is Tulki Ghuyrughi, since the bunch shape looks like a foxtail. Interesting to note, according to *V*IVC database Noyemberyani teghakan collected by us perfectly matched with ‘Lisyi Khvost’ (translated from Russian means foxtail) or ‘Tilki Kuyrugu’, ‘Telki kuruk’. However, the genetic profiles of Chilar and Noyemberyani teghakan were distinct and genetic relatedness was stated neither. Thus, Tulki Ghuyrughi can be considered as a case of homonymy. Probably the reason of confusion is related to the close morphology of bunch and berry shape. Both Chilar and Noyemberyani teghakan have oblong-cylindrical or cylindrical-conical, dense or loose, foxtail such as the shape of bunches and ovate berries. Genotype 16, whose identity could not be determined yet, is derived from a single plant. In the meanwhile, two further samples matching genotype 16 were recovered, proving its dissemination in former times.

The half-kinship relationships of Hadisi as a genitor were found with Sev Areni, Garan Dmak, Voskehat, Khatun khardji, Karmrahyut and Genotype 67. Respective Khatun khardji, an endangered autochthonous wine grape variety, full parentage resulted from a liaison of Hadisi and Vanqi, the latter being also an old Armenian variety. A PO relationship between Sev Areni and Sev khardji was determined. For the great part of autochthonous varieties, only single plants were recovered, indicating the threat of ancient cultivars and emphasizing the relevance of these surveys to safeguard the genetic diversity of indigenous varieties and to prevent them from extinction.

This group is encompassing also two representatives of the “Areni family”, namely Sev Areni and Mormor. During our surveys, 27 different clones were collected with matching genetic profiles and expressing unexpectedly high phenotype variability.

**Figure 3:**
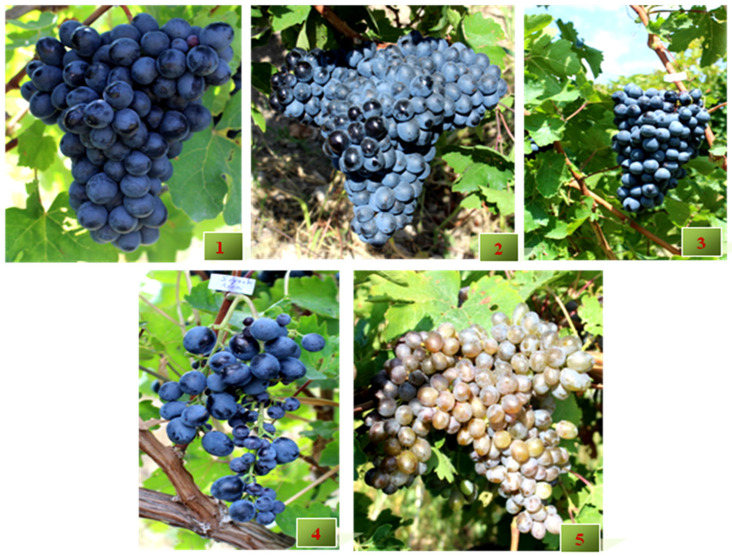
Bunch and berry morphology of Areni “family”: (1) Sev Areni. (2) Lyustra. (3) Movuz. (4) Seyrak Areni. (5) Mormor.

**Figure 4:**
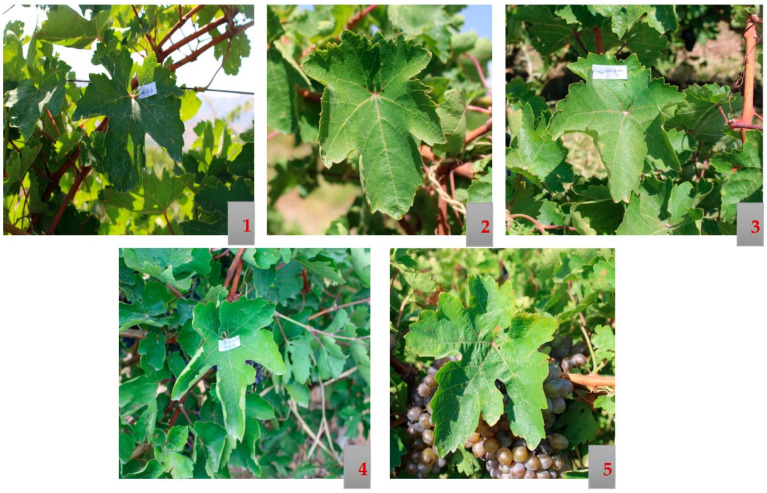
Leaves morphology of Areni “family”: (1) Sev Areni, (2) Lyustra. (3) Movuz. (4) Seyrak Areni. (5) Mormor.

The material was collected from different villages in the Vayots Dzor region, from vineyards older than 200 years. Sev Areni is the most iconic wine grape variety planted in Armenia for many centuries. Tumanyan described the variety in detail [44]. Until 1947 it was cultivated under the name Sev Malahi. Among the analyzed 27 genotypes Sev Areni, Seyrak Areni, Areni berqatu (Eghegnadzori № 4), Mormor and Kapuyt Mskhali are described in Armenian ampelographies as distinct varieties. The phenotypic descriptions in bibliography of these varieties perfectly matched with samples collected within the presented study. On Figure 3 and Figure 4 bunch, berry, and leaf morphology of Sev Areni, Lyustra, Movuz, Seyrak Areni, and Mormor are presented.

Movuz and Lyustra are not documented synonyms of Sev Areni in any of the Armenian ampelographies. While in the Caucasus and Northern black sea region ampelography for the Meleyi N. variety the authors noted Movuz, Urza sev and Areni Cherniyi as synonyms, which is a true case of homonymy. The major differences among these genotypes are associated with bunch shape and compactness, in the case of Mormor variety also with grey berry color. Mormor was described by Poghosyan as a neglected variety, cultivated in old vineyards in Vayots Dzor region [19]. The registered synonyms of Mormor are “Ampaguyn khaghogh” and “Mokhraguyn milagh”, means sky color or grey colored grape and local farmers used also “Sheklik Areni”, meaning “Blond Areni”. In fact, higher intravarietal variability was observed in “Areni family”. The most characterized polymorphisms leading to divergent phenotypes within varieties are associated with berry color locus. A somatic mutation affecting only one cell layer is leading to periclinal chimeras. Mormor is a chimeric variety and the stability of its chimeric structure is evidenced by the constant use of the variety during a long period.

As shown in Figure 3, obvious differences are related to bunch shape and density. Seyrak Areni has small, very loose, mainly conical or cylindrical-conical, sometimes winged bunches. In contrast, Lyustra shows dense or very dense, conical and winged bunches. Wings surround the bunch from all sides and due to this shape of the bunch, local farmers call it Lyustra, which means chandelier. Movuz was collected from different villages and vineyards. All samples displayed the same genetic profile matching Sev Areni.

Movuz and Sev Areni have medium-sized and conical-shaped, sometimes winged bunches and ovate/elliptic berries with a rounded top, covered by greyish-blue bloom. Movuz was identified as a clone of Sev Areni and thus the opinion that Movuz is a distinct variety was contradicted [67]. With respect to leaf morphology of Sev Areni, Lyustra, Movuz, Seyrak Areni, and Mormor, as well as other clones of Sev Areni, the most striking extraordinary trait is the involute leaf shape. The edges of the leaf blade are always folded upwards. Further ampelographic leaf characteristics are medium-sized, circular, sometimes slightly stretched, medium or deeply five-lobed leaves. The upper leaf surface is slightly wrinkled, glossy and hairless. The petioles are rich in anthocyanin. The main differences in leaf morphology are related to the shape of the teeth and the hairs on the lower side. The lower side of the leaves of true to type Areni is hairless, while the leaves of Seyrak Areni show bristle hairs on veins (Figure 4).

Interestingly, the group of unknown genotypes 16, 86, 95, 85, 93, 96, 31, 68, 70, and 55 clustered with endangered local wine grape Eraskheni and non-Armenian varieties Dunavski Lazur (Bulgaria) and Opsimos Edessis (Greece). Strikingly, seven unknown Genotypes (86, 95, 85, 96, 31, 68, and 70) showed female flower sex confirmed by APT3 marker analysis. The material was collected from very old, out of cultivation vineyards in Artsakh. Based on the morphology of berries (small, rounded, black berries) and bunch compactness (middle dense/loose) they were considered feral types. Further analysis is required to clarify the status of these samples. It is important to underline, that in the scope of the present study an unpredicted high number of female varieties, especially among autochthonous genotypes, were found. Being cross-pollinated, most likely they have played an important role in the formation of genetic diversity of Armenian grape germplasm. For Eraskheni involved in this group, first-order genetic relationships were not found. Eraskheni is a very old neglected wine and table grape variety described by Tumanyan and Melyan [44,50]. Some authors use the synonym of Eraskheni, Sev Urza, as a synonym of Sev Areni [49,68], which is again a case of homonymy.

The majority of Qishmish varieties were clustered involving ancient varieties Nazeli, Apoi khaghogh, Sev Qishmish, Marmari, Kishmish chernyi teinturier, Degin Yerevani, Vardaguyn Yerevani, Sermnali and Karmir Qishmish together with new bred cultivars Parvana, Arevar, Hrushaki, Ushahas Nazeli, Anush, and Zvartnots. Based on molecular fingerprinting the genetic profiles of old seedless varieties Marmari, Deghin Yerevani, and Vardaguyn Yerevani matched with Sultanina. Marmari/Kishmish mrarmornyi is a chlorophyllic mutant and Vardaguyn Yerevani/Kishmish rozovyii is a berry color mutant of Sultanina. Kishmish chernyi teinturier, considered as a berry flesh color mutant of Sev Qishmish/Kishmish chernyi variety. According to the parentage analysis, first-order relationships were found among Sultanina and Apoi khaghogh, Sermnali and Sev Qishmish varieties. According to the parentage analysis Sultanina as genitor of Nazeli was confirmed. Some authors referred Nazeli as new bred cultivar [69], which is not correctly interpreted, since Nazeli is one of the well-known old autochthonous raisin grape varieties described in detail by Tumanyan [44].

Two main groups were formed with new bred cultivars sharing common parents and unknown genotypes for which no genetic relationships were unraveled. Karmrahyut clustered with its offspring Arpa, Charentsi, Anushayut, Nerkarat, Merdzavan, Charentsi 2, and Artashati Karmir. The inferred Seyanets S 1212 was confirmed as the second parent. The usefulness of Colony software for reconstruction of genotypes, respectively, missing parents in pedigrees was demonstrated in previous studies as well [70,71]. Owing to the deduced progenitors (e.g., Angur Kalan, Muskat Hamburg and Karmrahyut), the following unknown genotypes 20, 21, 47, 61, 64, 65, and 72 were considered as new varieties. In spite of bibliographical studies, identification of these genotypes required clarification (Supplementary Table S5).

The neighbor-joining analysis grouped Muscat varieties, some new bred varieties, and unknown genotypes in Cluster II. Armenian new bred Muscat cultivars Muscat desertnyi, Muscat deghin, Muscat haykakan and named cultivar Muscat spitak Merdzavani were grouped with non-Armenian Muscat varieties Muscat Ottonel, Muscat à petits grains blancs and Muscat à petits grains noirs. The cluster involved also the variety Moschato Mavro from Greece, a progeny of Muscat à petits grains blancs and the true progenitor of Muscat haykakan. Armenian new cultivar Arazi has Chasselas musqué in its pedigree. The unknown Genotypes 57, 15, 24, 22, and 66 could not be identified and no relationships were found. Further clarifications related to the status of these samples are required.

## 5. Conclusions

Prospections in traditional viticulture regions across Armenia provided insights in the huge grapevine genetic diversity existing in the country. A combination of nuclear microsatellite markers and ampelography proved useful to determine the identity of collected samples recovered from old vineyards and home gardens. Synonyms, homonyms, alternative spellings, and misnomers were clarified. Well-identified and referenced grape genetic resources are a prerequisite for its utilization and the management of germplasm repositories. However, the assignment of variety names was not always successful. Sixty-seven genotypes could not be identified, due to missing genetic profiles in SSR databases or lack of names. Further bibliographical studies and cooperation with national germplasm repositories, preserving Armenian varieties is envisaged. First-degree genetic relationships between autochthonous varieties were uncovered. Missing parents might still exist in old vineyards but were not sampled yet or might have disappeared over time. Continuation of prospections to fill that gap is planned. The high number of new bred varieties included in the study reflect the enormous breeding activity in Armenia. Eleven non-determined genotypes were identified as new crosses due to the inferred parents involved in the cross. The high number of alleles, high observed and effective heterozygosity values, and presence of female APT3-allele 366, which is absent in western European cultivars, illustrate the huge diversity of Armenian germplasm. Presumably, these findings are related to recurrent introgression of *Vitis sylvestris* into the cultivated compartment during domestication events. Instability of grapevine cultivars was detected, showing three and in rare cases also four alleles at one locus. A deeper study of this quite frequent phenomenon will be carried out. So far, the present study is the first most representative and comprehensive analysis of Armenian grape germplasm. The majority of varieties is preserved non-grafted in the national grape germplasm collection at Etchmiadzin. Due to the recent arrival of Phylloxera in the Ararat valley, grafting on rootstocks is urgent and absolutely needed.

On the basis of realized in-depth investigation, a true-to-type inventory of Armenian grape germplasm was carried out and is documented in Armenian Vitis Database (www.vitis.am, 15 August 2021) and in Vitis International Variety Catalogue (www.vitis.de, 15 August 2021).

## Figures and Tables

**Table 1:** Descriptive statistics and genetic diversity of the 221 unique genotypes at 24 microsatellite loci.

Locus	Ra (bp)	Na	Ne	I	Ho	He	F
**VVS2**	123–155	15	8.454	2.279	0.864	0.882	0.021
**VVMD5**	226–266	13	6.521	2.032	0.853	0.847	−0.008
**VVMD7**	233–265	17	6.298	2.102	0.842	0.841	−0.001
**VVMD25**	237–269	11	4.973	1.731	0.796	0.799	0.003
**VVMD27**	176–198	12	4.991	1.788	0.837	0.800	−0.047
**VVMD28**	218–282	25	6.573	2.368	0.820	0.848	0.033
**VVMD3**2	232–292	19	4.891	2.084	0.827	0.796	−0.040
**VrZAG62**	180–206	13	6.799	2.105	0.846	0.853	0.008
**VrZAG79**	237–261	13	6.849	2.142	0.882	0.854	−0.033
**VVIv67**	338–401	22	5.039	2.184	0.761	0.802	0.050
**VrZAG67**	122–167	19	7.276	2.224	0.855	0.863	0.009
**VrZAG83**	180–201	6	2.990	1.224	0.692	0.666	−0.040
**VVIn1**6	147–157	5	3.037	1.272	0.673	0.671	−0.003
**VVIn73**	256–272	7	2.035	0.999	0.548	0.509	−0.076
**VVIp60**	306–332	13	3.820	1.741	0.645	0.738	0.126
**VVMD24**	204–220	10	4.129	1.664	0.787	0.758	−0.039
**VVMD21**	229–267	13	3.946	1.619	0.755	0.747	−0.042
**VMC4f3.1**	161–217	24	10.241	2.548	0.883	0.902	−0.011
**VVIb01**	285–319	12	3.006	1.381	0.732	0.667	−0.033
**VVIh54**	139–177	17	4.823	1.964	0.725	0.793	0.149
**VVIq52**	70–88	10	3.632	1.461	0.715	0.725	−0.073
**VVIv37**	144–178	19	8.788	2.369	0.864	0.886	−0.019
**VMC1b11**	167–205	17	5.449	2.029	0.782	0.816	−0.035
**VVIp31**	163–195	15	8.173	2.283	0.914	0.878	−0.099
**Total**		347					
**Min.**		5	2.035	0.999	0.548	0.509	−0.099
**Max**		25	10.241	2.548	0.914	0.902	0.149
**Mean**		14.485	5.531	1.900	0.787	0.789	0.000

Ra, range of allele size (bp); Na, number of different alleles; Ne, effective alleles; I, Shannon’s information index; Ho, observed heterozygosity; He, expected heterozygosity; F, fixation index.

**Table 2:** Replaced names of some of the autochthonous Armenian grapevine varieties.

New Name of Grapevine Variety	Old Name of Grapevine Variety
Ararati	Hachabash
Arevik	Alaghura
Vardaguyn Yerevani	Vardaguyn Qishmish
Karmir kakhani	Alakhki
Nazeli	Askyari
Voskehat	Khardji
Spitak Sateni	Spitak Khalili
Sev Arakseni	Sev Ezandari
Sev Areni	Sev Malahi
Sev Sateni	Sev Khalili
Arevar	Gyunei
Eraskheni	Sev Urza
Lalvari	Glglan, Danaburun
Koghbeni	Dava gyozi
Hastakot	Ayboghan
Hastamashk	Shirshira, Khozakashi
Chragi	Chrhagi
Nosrahat	Agha gyormaz
Spitak Areni	Spitak Malahi
Spitak Berri	Spitak Orduci Tchilar
Sev Aygeni	Ezan achq
Sevarbi	Karadali
Sevuk	Arabeni
Vagheni	Novrast
Vardabuyr	Gyulabi, Lalibedan

## Data Availability

Not applicable.

## Supplementary Materials

The following are available online at https://www.mdpi.com/article/10.3390/biology10121279/s1. Table S1. Primer sequences, chromosome positions, range, fluorophore, PCR multiplex and reference used in study; Table S2. MCPD data, assessment of trueness to type and bibliography of analyzed grapevine varieties; Table S3. List of few cases of identified varieties, new named, unknown varieties, synonyms and homonyms; Table S4. Microsatellite profiles for 24 nSSR loci of analyzed grapevine varieties and unknown genotypes; Table S5. Allele size (AS, bp) and allele frequency (AF) for the 24 nSSR markers; Table S6. Complete list of full parentages of analyzed varieties; Table S7. Complete list of half parentages of analyzed varieties.
